# Training interns in nutrition and dietetics: a cross-sectional study of the barriers and motivators to being a Registered Dietitian Nutritionist preceptor

**DOI:** 10.1186/s12909-021-02700-0

**Published:** 2021-05-16

**Authors:** Andrea M. Hutchins, Donna M. Winham, Jinette P. Fellows, Michelle M. Heer

**Affiliations:** 1 Department of Human Physiology and Nutrition, University of Colorado Colorado Springs, 1420 Austin Bluffs Parkway, Colorado Springs, CO 80918 USA; 2 Food Science & Human Nutrition, Iowa State University, 2302 Osborn Drive, Ames, IA 50011-1078 USA; 3 Department of Health Sciences, University of Colorado Colorado Springs, 1420 Austin Bluffs Parkway, Colorado Springs, CO 80918 USA; 4 Present Address: Clinical Dietitian, Colorado Mental Health Institute at Pueblo, 1600 W. 24th Street, Pueblo, CO 81003 USA

**Keywords:** Preceptors, Dietetics, Perceptions, Internship, Education, Beliefs, Attitudes, Motivators

## Abstract

**Background:**

As is common across the health professions, training of Registered Dietitian Nutritionists (RDNs) requires experiential learning for interns/students to gain skills and demonstrate entry-level competency. Preceptors are essential to the experiential learning component of health care professional training, providing supervision and mentoring as students and interns gain the skills required for entry-level practice competency. Over the past 27 years, 47–73% of applicants to dietetic internships have received a placement. Practitioners willing to volunteer as preceptors are needed to generate more internship or experiential learning opportunities for the profession to continue to meet workforce demands.

**Methods:**

The objective of this national-level online cross-sectional survey was to identify perceptions and attitudes associated with the preceptor role and incentives that might encourage precepting by current RDNs. A random sample of RDN and Nutrition and Dietetic Technicians, Registered (NDTR) professionals from the Commission on Dietetic Registration credentialed practitioner database were eligible to participate in the online survey. The main outcome measures included perceptions, attitudes, and preferred incentives to precept compared by preceptor experience categories (current, former, never precepted). Comparisons of perceptions, attitudes, and preferred incentives were made between preceptor experience categories using Chi-square and ANOVA.

**Results:**

Of 2464 invitations, 308 participants had complete variables for analysis. Top incentives were the opportunity to earn continuing education units (65.9%) and having expenses paid to attend a national conference (49.5%). Significantly more (*P* < 0.001) “former” and “never” preceptors reported the ability to choose when to take an intern, training on how to teach and communicate with interns, and access to an “on-call” specialist as incentives compared to “current” preceptors. Significantly more (*P* < 0.01) “never” preceptors reported training on internship expectations and the ability to provide input on intern selection process as incentives compared to “current” or “former” preceptors.

**Conclusions:**

Incentives to serve as a preceptor differ based on “current”, “former”, or “never” precepted status. Promoting and strategizing solutions to the current imbalance between the greater number of dietetic internship applicants compared to preceptors should be targeted based on preceptor status to retain current preceptors, encourage former preceptors to return and recruit professionals who have never served.

**Supplementary Information:**

The online version contains supplementary material available at 10.1186/s12909-021-02700-0.

## Background

As is common across the health professions, the successful preparation of Registered Dietitian Nutritionists (RDNs) and Nutrition and Dietetic Technicians, Registered (NDTRs) requires training in the practice setting to complement the didactic coursework. The United States (U.S.) Bureau of Labor Statistics projects an 8% growth rate in the number of jobs for RDNs from 2019 to 2029, 4% higher than the overall job growth rate in the U.S. [1]. Practicing in clinical, community, foodservice, and management settings as well as private practice, RDNs play a critical role in addressing the numerous health and health-related issues such as obesity, heart disease, diabetes, cancer, gastrointestinal disorders, and food security that have a nutrition component as well as working in a variety of foodservice and food industry roles.

There are multiple routes to meet the eligibility requirements for the registration exam for dietitians [2]. In the U.S., students may complete an accredited didactic program in dietetics and apply to an accredited dietetic internship or individualized supervised practice pathways program to obtain the required experiential learning. Students may also complete an accredited bachelor’s or graduate coordinated program in dietetics or a future graduate degree program [future education model (FEM) that is competency based], both of which combine the didactic coursework and experiential learning into one degree program. Therefore, dietetic internships, coordinated programs and FEM graduate programs all require experiential learning, or supervised practice, for the interns/students to gain the required skills and demonstrate entry-level competency in the field. Currently, about 70% of students who apply to dietetic internships following completion of a didactic dietetics program will secure a position [3]. However, the number of applicants securing an internship position has ranged from as low as 47% to as high as 73% over the past 27 years (1993–2019), and was 49% in 2015 when this study was conducted [4]. The routes to meet the eligibility requirements for the registration exam for nutrition and dietetic technicians include completion of an accredited associate’s degree program that includes didactic coursework and experiential learning, also requiring preceptors for supervision, or completion of a bachelor’s degree including an accredited didactic program in dietetics [5].

Practitioners who are willing to serve as preceptors, supervising interns during their experiential learning, are critical to this last training step required prior to the interns taking the RDN or NDTR credentialing exam. The supervision and mentoring provided by preceptors during the training of RDNs, NDTRs and other health care professionals is critical to students and interns gaining the skills required for entry-level practice competency. Those pursuing a career in nutrition and dietetics who are unable to secure a position to complete the required experiential learning may pursue other health professions or choose to practice as a non-RDN/NDTR nutritionist, reducing the number of credentialed RDNs/NDTRs available to fill open positions or provide counseling to clients as private practitioners. Practitioners willing to volunteer as preceptors are necessary to generate more internship or experiential learning opportunities to continue to meet workforce demand for credentialed RDNs/NDTRs.

While other health professions have explored the attitudes and perceptions associated with precepting, including the desired benefits and perceived barriers associated with the role [6–11], limited research has been conducted in the area of nutrition and dietetics despite the critical role preceptors play in the education and training of future RDNs and NDTRs [12–18]. Understanding what motivates practitioners to not only become preceptors, but to continue precepting, as well as the barriers to serving as a preceptor, is important if the dietetic profession is to successfully recruit sufficient preceptors now and in the future. The objective of this national study was to identify the perceptions and attitudes associated with the preceptor role and incentives that might encourage precepting among nutrition and dietetics professionals in the U.S.

## Methods

### Participants and recruitment

The target population for this national survey was RDN and NDTR professionals. In this cross-sectional study, the Commission on Dietetic Registration provided a list of 2,464 RDN and NDTR professionals that they randomly selected from their credentialed practitioner database. An email invitation with the online survey link was sent to the potential participants. The online questionnaire was completed via Survey Monkey (Palo Alto, CA) between August and September 2015. Two reminder emails were sent approximately 7–8 days apart. Participation was voluntary and completion of survey questions indicated consent. After completion of the survey, participants could choose to enter a drawing for a $25 Amazon.com gift card by providing an email address for the raffle via a separate webpage. One email was randomly selected for every 25 respondents who provided an email address. This study was approved by the University of Colorado Colorado Springs Institutional Review Board for the Protection of Human Subjects (IRB Protocol Number 16–005). All methods were carried out in accordance with relevant guidelines and regulations.

### Survey instrument development

The survey instrument was adapted from a previous published instrument [18]. During the development of the previous published instrument [18], 20 current DI preceptors, 5 non-preceptors, and 3 internship directors were asked about incentives and barriers associated with being a preceptor. They were also queried regarding interactions with interns. Themes identified from the interviews were developed into a survey for a formal pilot study with 24 current preceptors who did not participate in the initial interviews. Construct and content validity feedback from the pilot test respondents was used to refine the questionnaire used in the previous study [18]. For this study, adaptations to the previous published instrument were made to address limitations identified from the previous study [18], and the revised instrument was validated via external content review which included pretesting and post-test evaluation and feedback with four pilot respondents. Construct and content validity feedback from the validation testing for this study was used to further refine the revised questionnaire. Participant responses from the pilot test of the revised questionnaire used in this study were not included in the final survey analysis.

Survey questions assessed personal satisfaction, facility support, knowledge exchange, and interactions with the interns using a 5-point Likert scale, ranging from 1- strongly disagree, to 5 - strongly agree, to rank individual items. Participants were asked to rank incentives that could be offered for precepting as well as how they became aware of the opportunity to become a preceptor. Demographic information, such as employment setting, years of practice, age, gender, etc. were also gathered. The full questionnaire is available from the corresponding author.

### Data analysis and variable transformation

Cases with 10% or more variables for analysis missing were excluded from analysis. Descriptive and Likert-type question responses were compared by preceptor experience categories via Chi-square or ANOVA as appropriate [19]. The 5-category Likert-type questions were examined using principal components analysis with varimax rotation. The Kaiser-Meyer-Olkin measure confirmed sampling adequacy (.867). Eigenvalues and the scree plot suggested four underlying constructs [19]. Scale internal consistency or reliability was assessed by Cronbach’s alpha. Likert scales were created by summing the variable scores. All reported statistical tests except for the individual survey questions were two-sided with significance set at *P* < 0.05 using IBM SPSS Statistics for Windows, Version 26.0 (Armonk, New York, U.S.). All data generated or analyzed during this study are included in this published article (and its supplementary information files).

## Results

### Respondent characteristics

A total of 395 of the 2464 potential participants started the survey (e.g., clicked ‘begin survey’). Respondents who did not complete the survey (e.g., clicked ‘done’ at the end of the survey) were excluded (*n* = 41). Of the 354 respondents who completed the survey, respondents who did not indicate that they were an RD/RDN or DTR/NDTR (*n* = 22), who no longer worked in the nutrition field (*n* = 5), or who did not answer the question indicating their preceptor status (current, former, never precepted; *n* = 19) were excluded. Of the 332 respondents who indicated RDN or NDTR status, 322 (97%) were RDNs and 10 (3%) were NDTRs. The final analysis included 308 participants (12.5% of invited eligible participants), 300 (97.4%) RDNs and 8 (2.6%) NDTRs. Of the 308 participants analyzed, more participants had never served as a preceptor (41%) compared to those that were current preceptors (38%) or former preceptors (21%) (Table 1). Most respondents were non-Hispanic white, female, credentialed as an RDN, held a graduate degree, with a mean age of 44 ± 14.1 years (range 23–74). Former preceptors were significantly older than those who had never precepted and had been an RDN for more years (Table 1). The average years of practice were 19 ± 13 years with current and former preceptors having practiced for significantly more years than those who have never precepted. There were no differences in knowledge that non-RDNs could precept and almost all respondents were members of the Academy of Nutrition and Dietetics (the Academy). Over 97% of the participants were RDNs and more than half (56.5%) were employed in the clinical nutrition area.

**Table 1:** Distribution of demographic and credentialing characteristics of respondents by preceptor experience

Characteristics	Total (308)	Current Preceptors 38% (116)	Former Preceptors 21% (65)	Never Precepted 41% (127)
**Age in years (±SD**^a^)*	44 ± 14	44 ± 13^b, c^	48 ± 13^b^	42 ± 15^c^
**Years as an RDN (±SD;** ***n*** = 300)**	17 ± 13	17 ± 13 ^b, c^	21 ± 13 ^b^	14 ± 15 ^c^
**Years of Practice (±SD;** ***n*** = 292)**	19 ± 13	20 ± 13 ^b^	22 ± 13 ^b^	16 ± 13 ^c^
	
**Gender**				
Female	96.1	98.3	95.4	94.6
Male	3.9	1.7	4.6	5.4
**Self-reported “race”**				
White	92.5	90.5	92.3	94.5
Other	7.5	9.5	7.7	5.5
**Education**				
Bachelor’s degree or less	45.5	39.7	44.6	51.2
Master’s degree or more	54.5	60.3	55.4	48.8
**Credential status**				
Registered Dietitian Nutritionist	97.4	97.4	96.9	97.6
Nutrition and Dietetic Technician, Registered	2.6	2.6	3.1	2.4
**Employment Category**				
Clinical nutrition	56.5	58.6	60.0	52.8
Community or Government	21.4	24.1	15.4	22.0
Food Service – Retail	9.1	12.1	10.8	5.5
Private Practice/Consultant*	13.0	5.2^b^	13.8^c^	19.7^c^
**Know non-RDNs**^d^ **can precept**				
Yes	41.8	49.6	35.9	37.8
No	58.2	50.4	64.1	62.2
**Member of the Academy**^e^	99.7	99.1	100	100
**Emphasis on mentoring in training?**				
Yes	39.3	35.4	41.3	41.9
No	45.7	47.8	39.7	46.8
Don’t know/don’t remember	15.0	16.8	19.0	11.3

*P < 0.05; **P < 0.01

^a^*SD* Standard deviation

^b, c^Same superscript letters indicate column proportions that are not significantly different from each other

^d^*RDNs* Registered Dietitians Nutritionists

^e^the Academy = the Academy of Nutrition and Dietetics

Responses were received from 48 states with no respondents from Arkansas or Hawaii. Approximately 50% of the respondents came from 11 states (California, New York, Florida, Massachusetts, Michigan, Nebraska, North Carolina, Pennsylvania, Ohio, Texas, and Washington). Nine states had one respondent (Alaska, Connecticut, Delaware, Maine, Montana, North Dakota, South Dakota, Vermont, West Virginia) and four states had two respondents (Idaho, Kansas, Mississippi, and Wyoming).

Significant differences were observed by preceptor experience category for employment type with more respondents who were former preceptors or who had never precepted in private practice (13.8 and 19.7%, respectively) than current preceptors (5.2%) (Table 1). Respondents were asked if there was an emphasis in their classes or internship training on the importance of future mentoring as a professional obligation. Responses were not significantly different by preceptor experience category. The majority stated no (46%) or did not know or remember (15%).

### Attitudes and perceptions of the preceptor role and interns

#### Value of the preceptor role

Responses for attitude and perceptions of the value of the preceptor role are shown in Table 2. Current and former preceptors had significantly higher or more favorable scale scores for ‘professional duty to precept’. There was no significant difference by experience category for any other responses to questions about the value of the preceptor role. At least two-thirds of participants agreed with all the given reasons of value of the preceptor role except a lesser percentage (57%) agreeing that being involved with an internship gives prestige to the job.

**Table 2:** Response percentage for attitude and perceptions of value of preceptor role by preceptor experience category

Components of “Value of Preceptor Role” Scale Construct	Total (308)	Current Preceptor 38% (116)	Former Preceptor 21 (65)	Never Precepted 41% (127)
	
**Being a preceptor contributes to my profession**				
Disagree	1.0	0	0	2.4
Neutral	7.1	6.9	6.2	7.9
Agree	91.9	93.1	93.8	89.8
**Being a preceptor improves my teaching skills**				
Disagree	2.3	1.7	0	3.9
Neutral	8.4	9.5	1.5	11.0
Agree	89.3	88.8	98.5	85.0
**When I work with interns, I get a real sense of achievement**				
Disagree	1.0	0	1.5	1.6
Neutral	13.0	16.4	10.8	11.1
Agree	86.0	83.6	87.7	87.3
**Being a preceptor allows me to keep current and stimulated in my profession**				
Disagree	2.3	0.9	3.1	3.1
Neutral	11.7	12.9	6.2	13.4
Agree	86.0	86.2	90.8	83.5
**I believe I can be an effective preceptor**				
Disagree	2.9	0.9	3.1	4.7
Neutral	7.2	4.3	4.7	11.0
Agree	89.9	94.8	92.2	84.3
**I care about the fate of the dietetic internship program**				
Disagree	2.0	0.9	3.1	2.4
Neutral	7.5	5.2	3.1	11.9
Agree	90.6	94.0	93.8	85.7
**Involved internship gives prestige to job**				
Disagree	10.2	10.3	7.8	11.3
Neutral	32.6	30.2	39.1	31.5
Agree	57.2	59.5	53.1	57.3
Professional duty to precept***				
Disagree	5.9	5.2	3.1	8.1
Neutral	16.5	5.2^a^	14.1^b^	28.5^c^
Agree	77.6	89.7^a^	82.8^a^	63.4^b^

*** *P* < 0.001

^a, b, c^Same superscript letters indicate column proportions that are not significantly different from each other

#### Institutional support for preceptors

Responses for attitude and perceptions of institutional support for the preceptor role are shown in Table 3. Significantly more current preceptors agreed that there were adequate resources available to assist with intern training and their immediate supervisors are supportive of their preceptor role. Current and former preceptors agreed that the internship director schedules rotations at convenient times significantly more than those who had never precepted. Significant differences were found between the three precepting categories with the highest percentage of current preceptors agreeing that their immediate supervisors understand their role as a preceptor, followed by former preceptors and then those who had never precepted. Overall, the most common statement to which respondents across all precepting categories indicated they disagreed was ‘my workload is appropriate when I function as a preceptor’ (29%).

**Table 3:** Response percentage for attitude and perceptions of institutional support for preceptor role by experience category

Components of “Institutional support” Scale Construct	Total (308)	Current Preceptor 38% (116)	Former Preceptor 21% (65)	Never Precepted 41% (127)
	
Adequate resources are available to assist with intern training**				
Disagree	19.8	13.8^a^	19.4^a, b^	25.6^b^
Neutral	29.7	21.6^a^	32.3^a, b^	36.0^b^
Agree	50.5	64.7^a^	48.4^b^	38.4^b^
My immediate supervisors understand my role as preceptor*				
Disagree	7.0	5.2	4.8	9.8
Neutral	22.9	14.8^a^	23.8^a, b^	30.1^b^
Agree	70.1	80.0^a^	71.4^a, b^	60.2^b^
**My workload is appropriate when I function as a preceptor**				
Disagree	29.0	25.2	30.2	32.0
Neutral	28.1	22.6	27.0	33.6
Agree	42.9	52.2	42.9	34.4
Immediate supervisors supportive of my preceptor role***				
Disagree	4.6	0.0^a^	3.2^a, b^	9.6^b^
Neutral	20.9	9.6^a^	17.5^a^	32.^8b^
Agree	74.5	90.4^a^	79.4^b^	57.6^c^
The internship director schedules rotations at convenient times***				
Disagree	7.7	7.0	6.3	9.0
Neutral	39.0	25.2^a^	36.5^a^	53.3^b^
Agree	53.3	67.8^a^	57.1^a^	37.7^b^
**Intern activities have highlighted the functions of this department to administrators**				
Disagree	11.3	11.3	16.1	8.9
Neutral	39.7	40.0	48.4	35.0
Agree	49.0	48.7	35.5	56.1
**Precepting students increases awareness of my practice area or specialty**				
Disagree	5.3	4.3	7.9	4.8
Neutral	18.2	17.4	17.5	19.4
Agree	76.5	78.3	74.6	75.8

* *P* < 0.05, ** *P* < 0.01, *** *P* < 0.001

^a, b, c^Same superscript letters indicate column proportions that are not significantly different from each other

#### Perceptions of interns’ presence and value

Responses for attitude and perceptions of interns as a risk or asset at work are shown in Table 4. Significantly more current preceptors (86.0%) disagreed that interns may make serious mistakes and cause patient/client harm although most respondents across all precepting categories disagreed with this statement (78%). Significantly more current preceptors agreed that interns conduct themselves in a professional manner. Overall, most respondents disagreed to statements regarding interns as a risk and agreed to statements regarding interns as an asset. The most common intern as a risk statement to which respondents across all precepting categories indicated they agreed was ‘some interns are “know-it-alls”’ (18.5%). The most common intern as an asset statement to which respondents across all precepting categories indicated they agreed was ‘interns conduct themselves in a professional manner (83.8%).

**Table 4:** Attitude and perceptions regarding intern presence and value at work by preceptor experience category

	Total (308)	Current Preceptor 38% (116)	Former Preceptor 21% (65)	Never Precepted 41% (127)
	
***“Interns as a risk” Components***				
**I worry interns will contradict me and my teaching**				
Disagree	88.7	93.0	90.6	83.6
Neutral	9.3	7.0	7.8	12.3
Agree	2.0	0	1.6	4.1
Interns may make serious mistakes and cause patient/client harm*				
Disagree	78.0	86.0^a^	73.4^b^	73.0^b^
Neutral	18.3	11.4^a^	18.8^a, b^	24.6^b^
Agree	3.7	2.6	7.8	2.5
**Interns are difficult to relate to because of the generation gap**				
Disagree	85.5	85.2	82.8	87.1
Neutral	10.9	12.2	10.9	9.7
Agree	3.6	2.6	6.3	3.2
**Some interns are “know-it-alls”**				
Disagree	52.3	52.2	49.2	54.2
Neutral	29.2	31.3	23.8	30.0
Agree	18.5	16.5	27.0	15.8
***“Interns as an asset” Components***				
**Interns bring new ideas to department**				
Disagree	3.0	4.3	4.7	0.8
Neutral	18.5	15.7	18.8	21.0
Agree	78.5	80.0	76.6	78.2
**Intern projects conducted in this facility are useful to department**				
Disagree	5.0	3.5	6.3	5.7
Neutral	19.7	19.1	15.9	22.1
Agree	75.3	77.4	77.8	72.1
**Being a preceptor provides an opportunity to screen potential employees**				
Disagree	5.0	2.6	4.8	7.3
Neutral	22.8	20.0	17.5	28.2
Agree	72.2	77.4	77.8	64.5
Interns conduct themselves in a professional manner**				
Disagree	1.7	0.9^a^	6.3^b^	0^a^
Neutral	14.5	7.8^a^	17.2^a, b^	19.4^b^
Agree	83.8	91.3^a^	76.6^b^	80.6^b^

*P < 0.05, **P < 0.01

^a, b^Same superscript letters indicate column proportions that are not significantly different from each other

### Attitudes about the dietetic internship role scales

One-way ANOVA results are shown in Table 5. Two of the four scales were significantly different by preceptor category. Never preceptors had significantly lower scores for “value of the preceptor role” compared to current preceptors, but not in comparison to former preceptors. Current preceptors had a significantly more favorable score for “have support for precepting” compared to former preceptors or those who had never precepted. Correlations with the four scales and respondent age, years as an RDN, and years of practice were examined for possible confounding based on experience. Significant negative associations were found for age and value of the preceptor role (Pearson correlation coefficient (cc) -.108; *p* = .008), and for institutional support (cc − .119; *p* = .004). The number of years as a RDN and years practicing in nutrition showed similar significant associations for value of the preceptor role (Spearman’s rho cc − .192, *p* = .001) and institutional support (Spearman’s rho cc − .211, *p* < .001).

**Table 5:** Mean response values for Likert scale constructs by preceptor experience status (*n* = 308)

Characteristics, mean (SD^a^)	Total (308)	Current Preceptors 29% (161)	Former Preceptors 20% (113)	Never Precepted 51% (278)
Value of the preceptor role**	33.1 ± 4.0	33.7^a^ ± 3.7	33.3^a, b^ ± 3.7	32.4^b^ ± 4.4
Institutional support for precepting***	25.4 ± 4.3	26.7^a^ ± 4.0	25.1^b, c^ ± 4.4	24.2^b, c^ ± 4.1
**Interns as valuable asset**	15.8 ± 2.2	16.1 ± 2.1	15.6 ± 2.3	15.5 ± 2.1
**Interns as a risk at work**	8.3 ± 2.4	8.0 ± 2.1	8.8 ± 2.7	8.3 ± 2.4

*P < 0.05; ***P < 0.001; ^a, b, c^ indicates means that are significantly different from each other

^**a**^*SD* Standard deviation

### Incentives and barriers to precept

Incentives that might entice respondents to be more likely to take an intern are shown in Table 6. Significantly more respondents who had never precepted indicated that training on the internship expectations and the ability to provide input on the intern selection process would make them more likely to take an intern. The ability to choose when to take an intern, training on how to teach and communicate with interns, and access to an “on-call” specialist for help or assistance with issues when they arise were incentives for significantly fewer current preceptors compared to former preceptors and those who had never precepted.

**Table 6:** Respondents who would be more likely to take an intern if they received specified incentive

Incentive Option	Total (***n*** = 308)	Current Preceptors 38% (116)	Former Preceptors 21% (65)	Never Precepted 41% (127)
	
**Continuing professional education units (CPEUs) for field**	65.9	66.4	61.5	67.7
**Expenses paid to attend a national conference**	49.7	57.8	44.6	44.9
Ability to choose when to take an intern(s)***	42.9	22.4^a^	47.7^b^	59.1^b^
**Pay for my time**	40.9	38.8	49.2	38.6
Training on the internship expectations**	39.0	27.6^a^	35.4^a^	51.2^b^
Training on how to teach and communicate with interns***	32.5	16.4^a^	27.7^b^	49.6^b^
**Official reduction in workload while intern there**	30.5	29.3	32.3	30.7
Access to an “on-call” specialist for help or assistance with issues when they arise***	20.8	9.5^a^	23.1^b^	29.9^b^
Ability to provide input on intern selection process**	20.8	11.2^a^	23.1^a^	28.3^b^

**P < .01, ***P < .001

^a, b^Same superscript letters indicate column proportions that are not significantly different from each other

The most appealing incentive, chosen by approximately 66% of the respondents, was receiving continuing professional education units followed by having expenses paid to attend a national conference (50%), and the ability to choose when to take an intern (43%). Overall, the least appealing incentives were access to an ‘on-call’ specialist for help or assistance with issues when they arise (21%) and ability to provide input on the intern selection process (21%).

An analysis of compensation and training by current or former preceptor experience categories is presented in Table 7. There were no significant differences between the preceptor categories. The statement with which the highest percentage of current and former preceptors agreed was “my responsibilities as a preceptor are clearly defined” (65.2%) followed by “I had adequate preparation for my role as a preceptor” (59.9%) and “interns cause an increase in my workload” (59.8%). Approximately 98% of current and former preceptors disagreed with the statement “I receive extra monetary compensation when I take interns”. Statements with which a majority of current and former preceptors disagreed also include, “I feel pressured to take interns by my supervisor(s)”, “being a preceptor improves my chances of promotion and advancement” and “the DI director/clinical coordinator is unavailable to help me develop in my role as preceptor” (73.4, 66.1 and 64.8%, respectively).

**Table 7:** Percentage of compensation and training by current or former preceptor experience categories

Incentive	Total (181)	Current Preceptor 64% (116)	Former Preceptor 36% (65)
	
**I receive extra monetary compensation when I take interns**			
Disagree	97.8	97.4	98.4
Neutral	1.1	0.9	1.6
Agree	1.1	1.7	0
**Interns cause an increase in my workload**			
Disagree	26.4	27.2	25.0
Neutral	13.8	14.9	11.7
Agree	59.8	57.9	63.3
**Being a preceptor improves my chances of promotion and advancement**			
Disagree	66.1	67.0	64.5
Neutral	27.1	27.0	27.4
Agree	6.8	6.1	8.1
**Do not have adequate time to perform my job while I precept**			
Disagree	49.6	48.7	43.5
Neutral	31.1	30.4	32.3
Agree	22.0	20.9	24.2
**Receive insufficient compensation for taking interns**			
Disagree	36.9	38.3	34.4
Neutral	33.5	35.7	29.5
Agree	29.5	26.1	36.1
**My responsibilities as a preceptor are clearly defined**			
Disagree	16.3	12.9	22.6
Neutral	18.5	20.7	14.5
Agree	65.2	66.4	62.9
**I had adequate preparation for my role as preceptor**			
Disagree	14.1	9.6	22.6
Neutral	26.0	27.8	22.6
Agree	59.9	62.6	54.8
**There are adequate opportunities for me to share information with other preceptors**			
Disagree	38.4	33.9	46.8
Neutral	29.4	31.3	25.8
Agree	32.2	34.8	27.4
**There are guidelines that clearly outline the responsibilities of the DI director/clinical coordinator in relation to my preceptor role**			
Disagree	20.3	19.1	22.6
Neutral	29.9	32.2	25.8
Agree	49.7	48.7	51.6
**The DI director/clinical coordinator is unavailable to help me develop in my role as preceptor**			
Disagree	64.8	68.4	58.1
Neutral	23.9	21.1	29.0
Agree	11.4	10.5	12.9
**I feel pressured to take interns by my supervisor(s)**			
Disagree	73.4	72.2	75.8
Neutral	14.1	15.7	11.3
Agree	12.4	12.2	12.9

## Discussion

This national random sample survey with RDN and NDTR professionals identified differences by preceptor experience category in attitudes and perceptions of the dietetic internship preceptor role, institutional support, intern value, and preferred incentives to precept. Despite the important role preceptors perform by supervising dietetic interns during their experiential learning, little research has been conducted examining these aspects of the precepting experience for the dietetics profession [12–18], although other health professions have explored the precepting experience [6–11, 20]. If the dietetics profession hopes to address the imbalance between didactic program graduates and available internship positions [3, 4], and address the needs of the workforce by keeping up with the growth rate of the number of jobs for RDNs [1], additional preceptors will be needed to increase experiential learning opportunities. Therefore, examining the motivations and barriers of dietitians to precepting, and identifying strategies to enhance the motivations and address the barriers, is crucial.

A 2014 study with Arizona RDNs, NDTRs and other nutrition professionals utilized the same survey tool used in the current national survey [18]. Consistent with the current study, in the Arizona study more non-preceptors than current or former preceptors wanted the benefits of preceptor training and input with the dietetic internship intern selection process. More non-preceptors than current and former preceptors were interested in access to an “on-call” preceptor specialist and input into when they were scheduled to take interns in the Arizona study [18], whereas in the current study these benefits were significantly greater for both former and never preceptors.

### Value of the preceptor role

Overall, respondents in this study reported they believed great value was placed on the preceptor role, however they also felt there was a low perceived prestige associated with serving in the role (Table 2). Although these results appear to be inconsistent, they may reflect a difference in internal (value placed on preceptor role) versus external (perceived prestige associate with serving as a preceptor) perceptions and values. Even though the participants placed value on the role, they perceived little prestige placed on the role by colleagues or other health professionals.

Almost 86% of the respondents in this study agreed that they viewed precepting as a professional duty with current and former preceptors considering the preceptorship as their professional duty more so than non-preceptors (Table 2). Similarly, Canadian dietitians viewed precepting as a professional responsibility [16]. In other studies [6–9, 12, 15, 16, 18], preceptors for medical and nursing students as well as dietetics interns in the U.S. and internationally commonly mentioned giving back to the profession as the main reason they serve as preceptors.

Other research has stated that RDN preceptors in clinical settings across the United States reported about twice as many benefits to mentoring interns than their non-preceptor peers [12]. Congruent with the results of this study, the top-rated benefits of preceptorship by both preceptors and non-preceptors for the healthcare professions reported by other researchers were knowledge obtained, skills gained, and staying current in the profession [6–9, 11, 12, 15, 16].

### Institutional support for preceptors

Current and former preceptors in this study felt internship directors scheduled interns at a convenient time, and their immediate supervisors understood their role as a preceptor (Table 3). More current preceptors reported they had adequate training resources compared to former preceptors or those who had never precepted. Adequate training for preceptors is an ongoing challenge and programs across the health professions are addressing the need by developing and expanding training programs for their preceptors [21–23]. More current preceptors compared to former preceptors or those who had never precepted felt that their immediate supervisor was supportive of their preceptor role. However, employer support and recognition of the importance of the precepting role beyond the immediate supervisor is vital, and has been recognized as both a benefit and a barrier to precepting by other healthcare professions [6, 8–10] Encouraging employers to recognize the increased workload associated with precepting and provide workload adjustments or recognition or incentives on annual evaluations to encourage precepting could be helpful when recruiting more preceptors [24, 25].

### Preceptor perceptions of interns

Overall, respondents in this study had a positive impression of interns with greater than 75% of the respondents agreeing with 3 of the 4 survey components that assessed interns as an asset (Table 4). However, the only statistically significant difference was current preceptors agreeing that interns conducted themselves in a professional manner. Consequently, the preceptors’ attitudes toward and perceptions of interns appear to be assets, rather than barriers, to precepting, a theme that is also reported in the medical and nursing health professions [6, 9, 10].

Most participants in this study disagreed with the four statements that assessed interns as a risk (Table 4). Statistically more current preceptors (86%) disagreed with the statement that “interns may make serious mistakes and cause patient/client harm” compared to former preceptors or those who had never precepted. The statement with which the highest percentage of respondents disagreed (88.7%) was “I worry interns will contradict me and my teaching” although there were no statistically significant differences between the precepting categories.

In contrast to the ‘interns as a risk’ or ‘interns as an asset’ results from this study (Table 4), 61% of dietitians in a study of Canada internship/university course directors stated they encounter one or more challenging interns on a yearly basis [26]. Top reasons students were considered challenging included issues related to professional conduct (poor attitude, bad behavior, does not know limitations) and learning (does minimum work, does not prioritize learning, assumes “teach me” mentality) [26]. In a study that examined the decline in pediatric community preceptor teaching for medical students [6, 10], supervising students who were disengaged or not interested in experiencing pediatric practice were cited as reasons that pediatrician preceptors had decreased their precepting time or stopped precepting altogether.

### Incentives that might encourage becoming a preceptor

Participants in this study indicated the most appealing incentives to take an intern were continuing education units, the greatest response for all precepting experience categories, and paid expenses for national conference attendance. More former and non-preceptors were interested in the ability to choose when to take an intern, training on how to teach and communicate with interns, and access to an “on-call” specialist for assistance with precepting issues. Although not directly assessed in this study, the lack of these incentives may have contributed to the reasons a former preceptor stopped serving as a preceptor. Offering these incentives to prospective preceptors may increase the likelihood that they will agree to precept and are relatively low-cost or no-cost options for dietetic internships, coordinated programs and future education model graduate programs.

Consistent with finding of this study, the greatest motivational incentive for taking on interns for both preceptors and non-preceptors reported by other studies examining precepting in nutrition and dietetics was to receive continuing professional education units (CPEUs) and having expenses covered for a national conference [12, 18]. In contrast, a reason some pediatrician preceptors reduced their time precepting or stopped serving as a preceptor was inadequate monetary compensation for their time [6, 10], indicating that desired benefits vary across different health professions. Studies also report that benefits of satisfaction and altruism were greater in preceptors than non-preceptors [6, 10, 12, 13] and compensation was greater in non-preceptors [6, 10, 13].

As of June 1, 2017 RDNs serving as preceptors may record up to 3 CPEUs per year, and 15 CPEUs on their 5-year continuing education cycle for precepting for dietetics students in an Accreditation Council for Education in Nutrition and Dietetics (ACEND) accredited dietetics program [27]. The Commission on Dietetic Registration (CDR) provides a free 8 CPEU Preceptor Training Program available online [28].

A recent 2017 survey conducted by Nutrition and Dietetic Educators and Preceptors (NDEP) [29], an organization within the Academy of Nutrition and Dietetics, stated 53% of preceptors were not aware of CPEUs for precepting and about 61% either did not complete the CDR Online Dietetics Preceptor Training or did not know about it. NDEP provides preceptors free resources including two webinars, Guide to Being and Effective Preceptor, Part 1 and 2, eligible for 1.0 CPEU each [23]. The NDEP Council is dedicated to retention and recruitment of preceptors and participate in recognition of Outstanding Preceptor Awardees with financial support to attend FNCE [30]. Increased marketing of these preceptor benefits, especially via social media routes, may increase the willingness of practitioners who have never precepted to become preceptors.

### Barriers to precepting

A common barrier to precepting across health professions appears to be time [6, 8–10, 13, 31, 32]. Time constraint, followed by workload stress, were the greatest challenges to precepting for clinical RDNs with being understaffed/budget cuts [12] the top reason for not acting as a preceptor. Preceptors in the nursing and medical fields also identify time constraints, workload stress and perceptions of priority conflicts (e.g., patient vs. student) as challenges they face while precepting [6, 8–10]. In this study, many current and former preceptors reported an increase in their workload associated with precepting, and less than half of all respondents agreed that the workload is appropriate when they are serving as a preceptor. Increased workload demands and lack of recognition from peers and employers were barriers for precepting in dietetics, medicine, and nursing in the U.S. as well as internationally [6, 8–10, 16].

Even though RDNs and NDTRs can serve as preceptors as soon as they are credentialed [33], AbuSabha et al. [12] reported being a new dietitian or being in a new position was the second most common reason for not acting as a preceptor for clinical RDNs. In the study by AbuSabha et al. [12], preceptors had approximately 5 more years of experience than non-preceptors. In this study, those who had never precepted were significantly (*P* < 0.01) younger and had been an RDN for fewer years than former preceptors. Those who had never precepted had also been practicing for significantly (*P* < 0.01) fewer years than current or former preceptors.

Other researchers have reported preceptors with three or more years of experience as a preceptor had higher self-efficacy in knowledge, skills and communication/interaction [13]. The results of other studies also found younger RDNs valued intangible rewards and the benefit of professional enhancement more than RDNs aged 35 and older [13]. Younger RDNs and non-preceptors were more likely to consider lack of support, negative experiences and lack of appreciation as barriers to serving [13]. These combined results suggest younger RDNs, or those in their first years of practice, may be an untapped resource to serve as preceptors. However, they may not be volunteering to serve in the precepting role due to lack of confidence in their own practice, therefore they are not confident in their ability to supervise/teach interns [12, 13]. The lack of confidence in their own knowledge and skills and its impact on their ability to supervise/teach interns was reported in a study of nursing graduates [8]. In this study, Macey, et al. [8] reported recent nursing graduates found it difficult to serve as preceptors in an intensive care unit since they were also still learning and improving their skills as a beginning practitioner. Therefore, although younger health care practitioners, especially in the dietetics field, may be an untapped preceptor resource, they may also require additional support in the form of mentoring to enable them to serve effectively in this role.

### Missed opportunities with private practice practitioners/consultants as preceptors

Significantly more former preceptors (13.8%) and those who had never precepted (19.7%) reported having a private practice or serving as a consultant compared to current preceptors (5.2%). Private practitioners/consultants appear to be an underutilized source of preceptors. Identifying and overcoming the barriers to precepting for these groups, especially those who have never served as a preceptor, is an important avenue to pursue to increase potential preceptors.

Nearly 98% of the current and former preceptors who participated in this study did not receive monetary compensation when they took interns and almost 30% felt they received insufficient compensation for taking interns. Nearly 60% of respondents agreed that interns cause an increase in workload and 22% agreed that they do not have adequate time to perform their job while precepting. While these factors are concerns for preceptors working for an employer, their importance is magnified when considering their impact on potential preceptors who are in private practice or serve as consultants. Loss of income potential, increased workload, and inadequate time to devote to their private practice or consulting has a more direct impact on these practitioners. Therefore, even though private practitioners/consultants may be an untapped resource for potential preceptors, addressing their issues and barriers to precepting may look different than the methods used to recruit RDNs and NTDRs who work for an employer.

### Need to communicate importance of preceptors to current students and interns

Approximately 40% of respondents agreed there was an emphasis on mentoring in their education and training and almost 61% said there was not an emphasis on mentoring, or they did not remember. Stressing the importance of preceptors to the profession and the training of future practitioners is critical to the continuation of the profession. A mentoring and preceptor focused competency, Competency for Registered Dietitian Nutritionists (CRDN) 2.15: Practice and/or role-play mentoring and precepting others, was introduced in the release of the *2017 Accreditation Standards for Nutrition and Dietetics Coordinated Programs (CP), Didactic Programs (DPD), Internship Programs (DI), Technician Programs (DT) and Foreign (FDE)* and *International (IDE) Dietitian Programs* by ACEND [33]. Increased student/intern exposure to the mentoring/precepting role, including the opportunity to practice mentoring/precepting as part of their training, may increase the confidence of newly credentialed RDNs. Increased confidence in their ability to mentor/precept students/interns may result in an increased interest in precepting among this population.

### Strengths of study

The respondents in this study represented a randomly chosen cross-section of current and former RDN and NDTR credentialed preceptors as well as practitioners who have never precepted. Responses were representative of the major dietetics practice areas and participants were distributed throughout 48 states. A validated survey was used to collect data. The average age of the respondents (44 ± 14 years) was close to the median age of 40 years reported for RDN and NDTR practitioners [34].

### Limitations of study

The respondents in this study compared to the demographics provided by the Commission on Dietetic Registration for credentialed RDNs and NDTRs during the comparable time period [35] included a slightly higher percentage of RDNs compared to NTDRs (97.4% vs. 94.5%) and percentage of females (97% vs. 94%), and a higher percentage of those who identified as White (92.5% vs. 81%). Therefore, the results may not represent the views and experiences of current credentialed practitioners who are NDTRs, men, or persons of color. The average age of the respondents (44 ± 14 years) suggests that the attitudes and perceptions of RDNs and NDTRs who have been practicing for 5 years or less may not be adequately represented. Almost all the respondents were members of the Academy, so the results may not represent the views and experiences of credentialed practitioners who are not members of the Academy or non-credentialed current, former, or never precepted eligible to precept. Although the survey contained three questions that addressed whether the participant’s place of employment trained or accepted interns, the survey did not directly ask if there was a dietetic internship program hosted at the participant’s place of employment. Therefore, the results may not reflect differences in the views and experiences of preceptors whose place of employment hosted an internship versus those who trained or accepted interns from multiple internships. The survey was conducted between August and September 2015 which may limit the representation of the views and experiences of current credentialed practitioners. ISPPs had only been offered for a few years at the time this survey was conducted so the impact of those internship options on the views and experiences of credentialed practitioners may not reflected in the results of this study. The first cohort of FEM programs was established in 2017–2018, following data collection for this study, so the impact of those programs on the views and experiences of credentialed practitioners is not reflected in the results of this study.

## Conclusions

Incentives to serve as a preceptor differ based on current, former, or never served as a preceptor status. Our results suggest promoting and strategizing solutions to the current imbalance between dietetic internship applicants and qualified preceptors should be targeted based on current, former, or never precepted status in order to retain current preceptors, encourage former preceptors to return to precepting and recruit professionals that have never served as preceptors.

### Applications

Emphasizing the free CPEUs available to preceptors may help with recruiting new preceptors. Development of additional, ongoing free CPEU opportunities for those serving as preceptors may help retain them as preceptors, or entice former preceptors to start precepting again.

Preceptor training should address the perceived burden of increased work time spent supervising interns and include strategies for decreasing the perceived burden. The training needs to include ways to approach their employer, when applicable, regarding methods to address or mitigate the increased time and workload. Information for preceptors’ employers that addresses the issue of the time and workload involved in supervising interns could encourage increased support for precepting. Communication of precepting as an essential function of an RDN’s position and the prestige associated with training the next generation of practitioners to preceptors’ employers should be a function of both the Academy, ACEND, and the directors of supervised practice programs.

Encouraging both didactic and supervised practice programs to emphasize the critical role of preceptors in dietetics education and training to students and interns is essential. Promoting the importance of serving as a mentor or preceptor after obtaining the RDN or NDTR credential to support the profession is vital. Informing students and interns about the free CPEUs available to preceptors may encourage them to serve as a preceptor when eligible. Asking students to reflect on preceptors that had good time management while precepting can aid in the development these skills.

Based on the data from this study, utilization of practitioners who are in private practice is a missed opportunity. Development of programs that enhance the outreach to private practitioners by dietetic program directors and students/interns seeking supervised practice opportunities (e.g., for distance dietetic internships) could increase the number of preceptors from this area of practice. Exploring virtual rotation opportunities may also expand opportunities for students/interns to work with private practitioners even when proximity is a challenge.

### Opportunities for future research

Given the need to increase the diversity of practitioners as well as preceptors in the profession, additional research focusing on the attitudes and perceptions of those underrepresented (e.g., men, persons of color, transgender, non-binary) is needed. Research is also needed to address the time constraints and workload issues associated with serving as a preceptor.

## Supplementary Information

**Additional file 1.** Use of Preceptor Survey.

**Additional file 2.** Supplementary Data File.

## Data Availability

All data generated or analyzed during this study are included in this published article (and its supplementary information files).

## Abbreviations

ACEND: Accreditation Council for Education in Nutrition and Dietetics
ANOVA: Analysis of Variance
CDR: Commission on Dietetic Registration
CP: Coordinated Program
CPEUs: Continuing Professional Education Units
CRDN: Competency for Registered Dietitian Nutritionists
DI: Dietetic Internship Programs
DPD: Didactic Program in Dietetics
DT: Dietetic Technician Programs
FDE: Foreign Dietetic Education Programs
FEM: Future Education Model
IDE: International Dietetic Education Programs
IRB: Institutional Review Board
NDEP: Nutrition and Dietetic Educators and Preceptors
NDTR: Nutrition and Dietetic Technician, Registered
RDN: Registered Dietitian Nutritionists
SPSS: Statistical Package for the Social Sciences
U.S.: United States
