# Supplementary material for: Training interns in nutrition and dietetics: a cross-sectional study of the barriers and motivators to being a Registered Dietitian Nutritionist preceptor
Source: BMC Med Educ. 2021 May 16;21:277. doi: 10.1186/s12909-021-02700-0 (PMC8126131; doi:10.1186/s12909-021-02700-0)
Supplement: Supplementary file 1 — Additional file 1. Use of Preceptor Survey. [file 12909_2021_2700_MOESM1_ESM.pdf]

Training Interns in Nutrition and Dietetics: A Cross-Sectional Study of the Barriers and Motivators to Being a Registered Dietitian Nutritionist Preceptor

Authors

Andrea M. Hutchins, PhD, RD, FAND

Associate Professor, Department of Human Physiology and Nutrition, University of Colorado Colorado Springs, 1420 Austin Bluffs Parkway, Colorado Springs, CO 80918; Email: Andrea.Hutchins@uccs.edu

Donna M. Winham, DrPH, RD, FAND

Assistant Professor, Food Science & Human Nutrition, Iowa State University, 2302 Osborn Drive, Ames, IA 50011-1078; E-mail: dwinham@iastate.edu

Jinette P. Fellows, BS, RD

Affiliation at time research was completed: Undergraduate Student, Department of Health Sciences, University of Colorado Colorado Springs, 1420 Austin Bluffs Parkway, Colorado Springs, CO 80918

Current Affiliation: Clinical Dietitian, Colorado Mental Health Institute at Pueblo, 1600 W. 24th Street, Pueblo, CO 81003; Email: Jinette.Fellows@state.co.us

Michelle M. Heer, BS, RDN

Research Assistant, Food Science & Human Nutrition, Iowa State University, 2302 Osborn Drive, Ames, IA 50011-1078; Email: mmheer@gmail.com

Corresponding Author

Andrea M. Hutchins, PhD, RD, FAND, Associate Professor, Department of Human Physiology and Nutrition , University of Colorado Colorado Springs, 1420 Austin Bluffs Parkway, Colorado Springs, CO 80918, 719-255-4467, **Andrea.Hutchins@uccs.edu**

Use of Preceptor Survey

We are pleased to provide the Preceptor Survey we developed as part of this study. This questionnaire explores the perceptions and attitudes associated with the preceptor role and incentives that might encourage precepting among nutrition and dietetics professionals in the U.S. If you would like to use all or parts of this survey as part of your research, please review the following expectations related to its use.

- Proper acknowledgement of use of the instrument is provided in publication text as well as including the original reference in publications resulting from research that utilizes this questionnaire.
- The questionnaire as developed by the authors was developed and validated for the specific population reported in this study and has not fully validated for any other population.

DI intern mentor survey - National (7-13-15)

Hello!

If you work in the nutrition field or are a nutrition professional, we really want your opinion on training of dietetics students!

After completion of the survey, you will be eligible to enter a raffle for a $25 gift card to Amazon.com. One name will be drawn at random for every 25 completed surveys. The survey takes about 10-20 minutes to complete.

The survey purpose is to get your perspective of dietetic intern mentors. Preceptors or mentors serve as role models for students. They provide information, facilitate learning, and assess learning, among other responsibilities.

Please respond even if you are NOT a preceptor and feel you know little about being a mentor for interns. Your answers are confidential and will be reported only as grouped data.

Thank you in advance for your participation! Your opinions and views are essential to the success of this project. More detailed study information is on the next page. Please complete the survey by August 17, 2015.

Sincerely yours,

Jinette Fellows, nutrition student
Andrea M. Hutchins, PhD, RD
University of Colorado Colorado Springs

Approved UCCS Institutional Review Board Information Letter

University of Colorado
Colorado Springs (UCCS)
Consent to be a Research Subject

Title: Attitudes and Perceptions of the Dietetic Internship Preceptor Role by Nutrition Professionals in the United States.

Principal Investigator: Andrea M. Hutchins, PhD, RD

Introduction: If you work in the nutrition field or are a nutrition professional, we would like your opinion on the training of dietetics students.

Study Overview: We are conducting a research study to get your view of the role of mentors or preceptors in training dietetic student interns. We invite you to participate in this online survey, which will take about 10-20 minutes to complete. Please respond even if you are NOT a mentor for dietetic interns and feel you know little about being a mentor. It is important that we hear from people who are not involved in this process.

The survey purpose is to get your perspective on mentoring dietetic interns. Preceptors or mentors serve as role models for students. They provide information, facilitate learning, and assess learning, among other responsibilities.

Other people in this study : Up to 1,000 people will participate in this study.

Risks and Discomforts: Your participation in the study does not involve any foreseeable risks or discomforts. If you do not wish to answer any question, you may skip it and go to the next question. You have the option to withdraw from the study at any time.

Benefits: There will be no direct benefit to you by your participation in this research study; however, your opinions and views are important to the success of this project. Your responses to the survey will help us determine ways to make mentoring and training dietetic interns more beneficial for both the preceptor and the intern.

Compensation: After completion of the survey, you will be eligible to enter a drawing for a $25 Amazon.com gift card. If you choose to enter in the drawing you will be prompted to provide an email address at the end of the survey. Your email address, should you choose to provide it, will not be linked with your survey responses. One email will be selected for every 25 respondents while the survey is open for data collection (approximately 2 weeks). Your odds of winning a $25 gift card are 1 out of 25.

Confidentiality: The researchers will receive your responses anonymously. All data will be stored in a password protected electronic format and kept private. Any information that will make it possible to identify you as a participant (e.g., you provide your email address for the drawing) will be kept separate from your survey responses and destroyed at the conclusion of the study

Certain offices and people other than the researchers may have access to study records. Government agencies and UCCS employees overseeing proper study conduct may look at your study records. These offices include the UCCS Institutional Review Board, and the UCCS Office of Sponsored Programs and Research Integrity. UCCS will keep any research records confidential to the extent allowed by law. A study number rather than your name will be used on study records wherever possible. Study records may be subject to disclosure pursuant to a court order, subpoena, law or regulation.

Voluntary Participation and Withdrawal from the Study: Taking part in this study is voluntary. You have the right to leave the study at any time without penalty. You may refuse to answer any survey questions you do not feel comfortable with. If you withdraw from the study, you may request that your research information not be used by contacting the Principal Investigator listed above and below.

Contact Information

Contact (PI's info): Andrea.Hutchins@uccs.edu

- if you have any questions about this study or your part in it,
- if you have questions, concerns or complaints about the research, or
- if you would like information about the survey results when they are prepared.

Contact the Research Integrity Specialist at 719-255-3903 or via email at irb@uccs.edu:

- if you have questions about your rights as a research participant, or
- if you have questions, concerns or complaints about the research.

Electronic Consent

Please print a copy of this consent form for your records, if you so desire.

I have read and understand the above consent form, I certify that I am 18 years old or older and, by clicking the <Next> button to enter the survey, I indicate my willingness voluntarily take part in the study.

DI intern mentor survey - National (7-13-15)

Respondent gender and age

First, please tell us a bit about you:

* 1. Are you currently a full-time undergraduate or graduate student? (This question requires a response to continue.)

- Yes
- No
- Prefer not to answer

* 2. Please select your gender. (This question requires a response to continue.)

- Female
- Male
- Prefer not to answer

3. In which year were you born?

Please select a year from the drop down menu below or write in below if not shown.

Other (optional)

DI intern mentor survey - National (7-13-15)

Demographic Information

4. Are you of Hispanic, Latino, or Spanish origin?

- ○ No, not of Hispanic, Latino, or Spanish origin
- ○ Yes, Mexican, Mexican American, Chicano
- ○ Yes, Puerto Rican
- ○ Yes, Cuban
- ○ Yes, another Hispanic, Latino, or Spanish origin

If other, please specify

5. Which one or more of the following would you say is your race?

CHECK ALL THAT APPLY

- ☐ African American, Black, Afro-Caribbean
- ☐ American Indian or Alaskan Native
- ☐ Asian Indian
- ☐ Chinese
- ☐ Japanese
- ☐ Korean
- ☐ Native Hawaiian or other Pacific Islander
- ☐ White
- ☐ Other race(s)

If other, please specify

6. Please enter your 5 digit zip code below.

ZIP:

Education

7. What is the highest level of education that you have completed?

- 11th grade or less
- Completed GED
- High school diploma
- Some college credit, but less than 1 year
- 1 or more years of college, no degree
- Associate degree (ex: AA or AS)
- Bachelor’s degree (BA, BS, AB)
- Master’s degree (MS, MA, MEd, MBA)
- Doctorate degree (PhD, DrPH, ScD)

Other (optional)

Academy membership and credentialing

8. Are you a member of the Academy of Nutrition and Dietetics (The Academy or AND)?

- Yes
- No
- Former Academy member, not currently

Other (please specify)

9. Do you have any of the following credentials?

You may select all that apply.

- Registered Dietitian (RD) or Registered Dietitian Nutritionist (RDN)
- Dietetic Technician Registered (DTR)
- Certified Dietary Manager (CDM)
- Board Certified Specialist in Pediatric Nutrition
- Board Certified Specialist in Sports Dietetics
- Board Certified Specialist in Gerontological Nutrition
- Board Certified Specialist in Oncology Nutrition
- Board Certified Specialist in Renal Nutrition
- Fellow of the Academy of Nutrition and Dietetics
- Fellow of the American Dietetic Association
- Certified Nutrition Support Specialist
- Board Certified Advanced Diabetes Management (BC-ADM)
- Certified Diabetes Educator (CDE)
- None of the Above

Other Please Specify

DI intern mentor survey - National (7-13-15)

Your RD/RDN training experience

10. How did you complete the necessary training to become a Registered Dietitian/Registered Dietitian Nutritionist?

- Regular internship
- Individualized Supervised Practice Pathway (ISPP)
- Combined Masters/internship program
- Masters plus 6-months work experience
- Coordinated DPD/internship program
- 'Grandfathered' RD when requirements changed
- Have not completed necessary training to become a RD/RDN
- Other (please explain below)

Explanation

11. How many years have you been an RD?

Please select number of years from the drop-down menu

Professional service as part of your training

12. Think back to your training in dietetics. Was there emphasis in your classes or internship on giving back to the profession through mentoring?

- Yes
- No
- Don't know/don't remember

Other (please specify)

Current employment setting

13. What is your primary employment setting? Please select the one that best fits.

- Hospital (in-patient/acute care)
- Clinic or Ambulatory care center
- Extended care facility
- Healthcare provider (HMO, physician)
- Community/Public Health program
- Government agency (Federal, State, or County)
- School food service (elementary,high school, college)
- Faculty member at college or university
- Food manufacturer/distributor/retailer
- Supermarket
- Consultant - consumer
- Consultant - health care facility
- Consultant - corporate
- Research
- Private practice

Other (optional)

Job title

14. What is your main job title?
(Select one only)

- ○ Clinical Dietitian
- ○ Clinical Nutrition Manager
- ○ Registered Dietitian/Registered Dietitian Nutritionist
- ○ Dietetic Technician
- ○ Dietary Coordinator
- ○ Food Service Director
- ○ Faculty
- ○ Nutrition Consultant
- ○ Vendor Representative
- ○ Public Health Dietitian
- ○ Dietetic Researcher
- ○ Pediatric Dietitian
- ○ Neonatal Dietitian
- ○ Gerontological Dietitian
- ○ Diabetes Educator

Other (please specify)

Nutrition and dietetics experience

From the Academy of Nutrition and Dietetics (AND) website, the following definition of dietetics is provided:

"Dietetics is defined as the integration and application of principles derived from the sciences of nutrition, biochemistry, physiology, food management and behavioral and social sciences to achieve and maintain people's health."

15. Are you currently employed in the nutrition and dietetics field?

- Yes
- Yes, self-employed
- No, employed in another field
- Not currently employed
- Maybe, not sure
- Other (please specify)

16. If applicable, how many total years have you worked in the nutrition and dietetics field?

Please select an option from the drop down menu below.

OPTIONAL: Comments

DI intern mentor survey - National (7-13-15)

Eligibility to be a DI preceptor/mentor

Dietetic intern mentors or preceptors serve as role models for students during their dietetic internship experience. They provide information, facilitate learning, and assess learning, among other responsibilities.

Dietetic internship preceptors/mentors do not have to be RDs, RDNs, DTRs, or other certified nutrition or dietetics professionals. Other qualified personnel with 1 year or more of professional experience may be eligible to precept/mentor interns.

In fact, many nutrition professionals provide intern education opportunities in non-traditional dietetic settings such as product development, pharmaceutical or supplement sales, culinary settings, spas, gyms, corporate wellness, and research.

17. Did you know that DI preceptors/mentors could be other professionals besides RDs/RDNs and DTRs?

- ○ Yes
- ○ No

Preceptor/mentor status

18. Does your current place of employment train dietetic interns?

- Yes
- No
- Do not know
- I am currently unemployed

Other (optional)

19. Do you personally serve as a Dietetic Internship preceptor/mentor now?

- Yes
- No
- Not at this time, but I have in the past

Comments

Current or past preceptors

20. Which option below best describes your role as a dietetic intern preceptor now?

- Dietetic Internship Preceptor
- Director - Dietetic Internship
- Former Dietetic Internship Preceptor
- Former Director- Dietetic Internship
- No involvement with dietetic interns

Other (please specify)

Preceptor characteristics

21. How many total years have you mentored dietetic interns?
OR For how many years did you precept interns if you are no longer a preceptor?

Please select an option from the drop down menu

OPTIONAL: Comments

22. How many dietetic interns receive(d) training at your facility each year?

- 0
- 1-2
- 3-4
- 5-6
- 7-8
- 9-10
- 11 or more
- I do not know

Other (please specify)

Average time with interns

23. On average, how many weeks per year do YOU (or did you) have contact time with each intern?

Please select an option from the drop down menu below.

Comments

DI intern mentor survey - National (7-13-15)

Perceptions of being a Dietetic Internship preceptor for current or past preceptors

The following sections ask about the positive and negative perceptions of being a dietetic internship preceptor. Based on previous published research, qualitative interviews, and pilot testing, several themes emerged as important barriers and/or motivators for successful precepting.

These included: personal satisfaction, knowledge exchange, facility support,and interactions with the interns. Please tell us what you think.

Personal satisfaction, self-efficacy, and knowledge

24. Please check the ONE selection for each statement that comes closest to reflecting your opinion about it.

	Strongly disagree	Disagree	Neutral	Agree	Strongly agree	I have never been a preceptor
When I work with interns, I get a real sense of achievement	○	○	○	○	○	○
Being involved in an internship program adds prestige to my job	○	○	○	○	○	○
I believe it is a professional duty to precept	○	○	○	○	○	○
Being a preceptor improves my teaching skills	○	○	○	○	○	○
Being a preceptor allows me to keep current and stimulated in my profession	○	○	○	○	○	○
Being a preceptor contributes to my profession	○	○	○	○	○	○
I believe I can be an effective preceptor	○	○	○	○	○	○
Agreeing to be a preceptor was a definite mistake on my part	○	○	○	○	○	○
I care about the fate of the dietetic internship program	○	○	○	○	○	○

OPTIONAL: Comments

Facility support for Dietetic Internship Preceptors

25. Please check the ONE selection for each statement that comes closest to reflecting your opinion about it.

	Strongly disagree	Disagree	Neutral	Agree	Strongly agree	I have never been a preceptor
My immediate supervisors are supportive of my role as preceptor	◯	◯	◯	◯	◯	◯
The facility administration is NOT supportive of the internship program	◯	◯	◯	◯	◯	◯
My immediate supervisors understand my role as preceptor	◯	◯	◯	◯	◯	◯
My workload is appropriate when I function as a preceptor	◯	◯	◯	◯	◯	◯
Adequate resources are available to assist with intern training	◯	◯	◯	◯	◯	◯
The internship director schedules rotations at convenient times	◯	◯	◯	◯	◯	◯
Intern activities have highlighted the functions of this department to administrators	◯	◯	◯	◯	◯	◯
Precepting students increases awareness of my practice area or specialty	◯	◯	◯	◯	◯	◯
Being a preceptor provides an opportunity to screen potential employees and job recruit	◯	◯	◯	◯	◯	◯

OPTIONAL: Comments

Compensation and training for Dietetic Internship preceptors

Please check the option that best describes your feelings to the following statements. If you are not currently a preceptor, think back to the last time that you interacted with interns.

26. Please check the ONE selection for each statement that comes closest to reflecting your opinion about it.

	Strongly disagree	Disagree	Neutral	Agree	Strongly agree	I have never been a preceptor
I receive extra monetary compensation when I take interns	○	○	○	○	○	○
Interns cause an increase in my workload	○	○	○	○	○	○
Being a preceptor improves my chances of promotion and advancement	○	○	○	○	○	○
I do not have adequate time to perform my job responsibilities while I function as a preceptor	○	○	○	○	○	○
I receive insufficient compensation for taking interns	○	○	○	○	○	○
My responsibilities as a preceptor are clearly defined	○	○	○	○	○	○
I feel I had adequate preparation for my role as preceptor	○	○	○	○	○	○
There are adequate opportunities for me to share information with other preceptors	○	○	○	○	○	○
There are guidelines that clearly outline the responsibilities of the DI director/clinical coordinator in relation to my preceptor role	○	○	○	○	○	○
The DI director/clinical coordinator is unavailable to help me develop in my role as preceptor	○	○	○	○	○	○
I feel pressured to take interns by my supervisor(s)	○	○	○	○	○	○

OPTIONAL: Comments

The dietetic interns themselves

27. Please check the ONE selection for each statement that comes closest to reflecting your opinion about it.

	Strongly disagree	Disagree	Neutral	Agree	Strongly agree
My workplace or situation would not allow interns					
There is not enough for interns to do at my workplace					
Intern projects conducted in this facility are useful to the department					
Interns bring new ideas to our department					
Interns conduct themselves in a professional manner					
Intern projects decrease my workload					
Interns do not dress properly for the workplace setting					
Some interns lack the necessary technical skills and knowledge					
Interns are difficult to relate to because of the generation gap					
I worry interns will contradict me and my teaching					
Some interns are 'know-it-alls'					
Interns may make serious mistakes and cause patient/client harm					

OPTIONAL: Comments

Satisfaction with being a DI preceptor

28. If it were completely up to you, would you stop precepting dietetic interns?

- Yes, I would stop taking interns
- No, I would continue to take interns
- Maybe, not sure

Optional comment

Interest and knowledge of mentoring interns

29. Have you ever thought about becoming a dietetic internship preceptor/mentor?

- Yes
- No

Other (please specify)

30. Are you aware of how to become a dietetic internship preceptor/mentor?

- Yes
- No

Other (please specify)

DI intern mentor survey - National (7-13-15)

Perceptions of being a Dietetic Internship Preceptor

The following sections ask about the positive and negative perceptions of being a dietetic internship preceptor/mentor. Based on previous published research, qualitative interviews, and pilot testing, several themes emerged as important barriers and/or motivators for successful precepting/mentoring.

These included: personal satisfaction, knowledge exchange, facility support,and interactions with the interns. Please tell us your opinions on these statements.

Personal satisfaction, self-efficacy, and knowledge

31. Please check the ONE selection for each statement that comes closest to reflecting your opinion about it.

	Strongly disagree	Disagree	Neutral	Agree	Strongly agree
If I worked with interns, I would get a real sense of achievement	○	○	○	○	○
Being involved in an internship program would add prestige to my job	○	○	○	○	○
I believe it is a professional duty to precept	○	○	○	○	○
Being a preceptor would improve my teaching skills	○	○	○	○	○
Being a preceptor would allow me to keep current and stimulated in my profession	○	○	○	○	○
Being a preceptor would contribute to my profession	○	○	○	○	○
I believe I could be an effective preceptor	○	○	○	○	○
Agreeing to be a preceptor would be a definite mistake on my part	○	○	○	○	○
I care about the fate of the dietetic internship program	○	○	○	○	○

OPTIONAL: Comments

Facility support for Dietetic Internship Preceptors

32. Please check the ONE selection for each statement that comes closest to reflecting your opinion about it.

	Strongly disagree	Disagree	Neutral	Agree	Strongly agree
My immediate supervisors would be supportive of my role as preceptor	◯	◯	◯	◯	◯
The facility administration would NOT be supportive of the internship program	◯	◯	◯	◯	◯
My immediate supervisors would understand my role as preceptor	◯	◯	◯	◯	◯
My workload would be appropriate if I functioned as a preceptor	◯	◯	◯	◯	◯
Adequate resources would be available to assist with intern training	◯	◯	◯	◯	◯
The internship director would schedule rotations at convenient times	◯	◯	◯	◯	◯
Intern activities would highlight the functions of this department to administrators	◯	◯	◯	◯	◯
Precepting students would increase awareness of my practice area or specialty	◯	◯	◯	◯	◯
Being a preceptor would provide an opportunity to screen potential employees and job recruit	◯	◯	◯	◯	◯

OPTIONAL: Comments

The dietetic interns themselves

33. Please check the ONE selection for each statement that comes closest to reflecting your opinion about it.

	Stongly disagree	Disagree	Neutral	Agree	Strongly agree
My workplace or situation would not allow interns	○	○	○	○	○
There would not be enough for interns to do at my workplace	○	○	○	○	○
Intern projects conducted in this facility would be useful to the department	○	○	○	○	○
Interns would bring new ideas to our department	○	○	○	○	○
Interns would conduct themselves in a professional manner	○	○	○	○	○
Intern projects would decrease my workload	○	○	○	○	○
Interns would not dress properly for the workplace setting	○	○	○	○	○
Some interns lack the necessary technical skills and knowledge	○	○	○	○	○
Interns would be difficult to relate to because of the generation gap	○	○	○	○	○
I worry interns would contradict me and my teaching	○	○	○	○	○
Some interns are 'know-it-alls'	○	○	○	○	○
Interns may make serious mistakes and cause patient/client harm	○	○	○	○	○

OPTIONAL: Comments

DI intern mentor survey - National (7-13-15)

Local vs. Distance Internships

Interns in a local internship program complete the internship at sites within the state, region, county and/or city in which they reside. The intern is required to live in the internship location area regardless of where the intern resides at the time of application. Internship directors identify the preceptors and location for each of the interns.

34. Does your facility (or you) precept interns from established local internships in your area?

- Yes
- No
- Do not know
- Not applicable

Comments

Local vs. distance internships

Interns in a distance internship program complete the internship where they live. Typically the applicant must locate and identify preceptors and supervised practice facilities prior to submitting the application. For these programs, the internship director does NOT usually assist with identifying preceptors or locations.

35. Does your facility (or you) accept interns from distance internship programs?

- Yes
- No
- Do not know
- Not applicable

Comments

Encouragement to precept

What types of incentives or changes would encourage you to mentor interns, or to mentor more interns?

36. I would be more likely to take an intern if I received ...

(Check all that apply)

- [ ] Continuing education units (CEUs) for my field
- [ ] Pay for my time
- [ ] Expenses paid to attend a national conference,e.g. FNCE, SNA
- [ ] Official reduction in my regular workload while intern there
- [ ] The ability to provide input on the intern selection process
- [ ] The ability to choose when to take an intern(s)
- [ ] Training on the internship expectations
- [ ] Training on how to teach and communicate with the interns
- [ ] Access to an "on-call" specialist for help or assistance with issues when they arise

Other (please specify)

DI intern mentor survey - National (7-13-15)

Liability for dietetic interns

Dietetic interns are required to carry personal liabilty insurance while they are in supervised practice settings.

37. Are you (or would you) be concerned about liability of dietetic interns under your supervision?

- Yes
- No
- Not sure

Other (please specify)

DI intern mentor survey - National (7-13-15)

Thank you for your input!

We thank you for your time and thoughtful responses in completing our survey. If you would like to be entered into a drawing to receive a $25 gift card to Amazon.com, please enter a valid email address on the next page. Your email address will not be associated with your answers to this survey. Remember - one name will be drawn at random for every 25 completed surveys!

When you click "done" for this survey, you will be automatically taken to a separate survey page that is a different color to enter your email information.
